# Cytomegalovirus reactivation enhances the virulence of a staphylococcus aureus pneumonia in a mouse model

**DOI:** 10.1186/2197-425X-3-S1-A831

**Published:** 2015-10-01

**Authors:** S Hraiech, J Bordes, JL Mège, X De Lamballerie, S Lehingue, C Guervilly, J-M Forel, D Raoult, L Papazian

**Affiliations:** Réanimation - Détresses Respiratoires et Infections Sévères, APHM, CHU Nord, Marseille, France; IHU Méditerranée Infection, URMITE CNRS IRD INSERM UMR 7278, Marseille, France; Fédération d'Anesthésie-Réanimation-Urgences-Surveillance Continue-Centre de Dialyse BCRM Toulon Hôpital d'Instruction des Armées Sainte-Anne Toulon, Toulon, France; Unite des Virus Emergents UMR 190 'Emergence des Pathologies Virales', Marseille, France

## Introduction

Cytomegalovirus (CMV) reactivation is common in immunocompetent mechanically ventilated patients. Lungs are a frequent site of reactivation. CMV reactivation may be associated with higher mortality among these patients [1]. Some studies have suggested that CMV reactivation may be associated with higher incidence of nosocomial pneumonia [2, 3].

## Objectives

The aim of this study was to assess the virulence of a staphylococcal pneumonia developed during CMV reactivation in a mouse model.

## Methods

The study was approved by our local ethic committee. Female BALB/c mice were used in all experiments. CMV primo-infection was obtained by intra-peritoneal inoculation of 2 x 10^4^ PFU of murine CMV (MCMV) Smith strain. Seropositivity was confirmed by immunofluorescence in serum. MCMV was considered to be latent 4 months later. Reactivation was triggered by cecal ligature and puncture. Mice were considered to have a CMV reactivation 2 weeks later [4]. After this, 20 MCMV positive mice underwent an intra-nasal inoculation with 5 × 10^8^ CFU of Staphylococcus aureus to induce pneumonia. Twenty MCMV negative BALB/c mice were treated according to the same protocol, including cecal ligature and puncture (control group). Daily weight, signs of sepsis and spontaneous mortality were noted. After 15 days, surviving mice were euthanized. Blood and lung were collected for bacterial culture and histological examination. MCMV reactivation was assessed by RT-PCR in lungs and salivary glands. A second cohort of 20 mice were treated according to the same protocol (10 MCMV positive and 10 MCMV negative mice) but were sacrificed at day 2 and day 5 after pneumonia.

## Results

No mortality from staphylococcal pneumonia was observed in the control group whereas the mortality rate was of 10 % in the MCMV group (p = 0.15). Mean weight loss at day 1 was higher in MCMV mice than in control (1.5 g versus 0.9 g respectively). Macroscopic observation and bacteriological analysis of lungs showed staphylococcal abscesses in 4/20 (20%) mice in MCMV group as compared to 0/20 in control group at day 15. At day 5, 3/5 mice had lung abscesses in MCMV group as compared to 0/5 in control group. No lung abscesses were present at day 2 after pneumonia. Overall, 7/30 (23%) mice had lung staphylococcal abscesses in MCMV group as compared to 0% in control group (p = 0,005). Mean lung bacterial count was significantly higher in MCMV mice as compared to control at day 2 (2.4 × 10^5^ vs. 2.4 × 10^2^ CFU/ lung, p = 0.009), day 5 (2.1 ×10^5^ vs. 5.5 × 10^2^ CFU/lung, p = 0.02) and day 15 (5.5 × 10^1^ vs. 0 CFU/lung, p = 0.04).

## Conclusions

In a mouse model, CMV reactivation leads to the switch from a non lethal to a lethal staphylococcal pneumonia, increases bacterial lung count and favors the occurrence of staphylococcal lung abscesses.
